# The association between internet use and cognition in older adults: empirical analysis based on CLASS 2023

**DOI:** 10.3389/fpsyg.2026.1801429

**Published:** 2026-05-08

**Authors:** Chunhui Yan, Peiyou Chen, Zhihao Jia, Guangwu Dong

**Affiliations:** 1 College of Sports Science, Nanjing Normal University, Nanjing, China; 2 College of Physical Education, Shihezi University, Shihezi, Xinjiang, China

**Keywords:** activities of daily living, China longitudinal aging social survey (CLASS), cognition, depression, internet use

## Abstract

**Objective:**

This study aims to investigate the relationship between Internet use and cognition among older adults and to elucidate the mediating mechanisms involving activities of daily living and depression.

**Methods:**

Utilizing data from the 2023 China Longitudinal Aging Social Survey (CLASS), a sample of 9,012 older adults aged 60 to 90 was selected. A chain mediation model was constructed and analyzed using the PROCESS macro (Model 6) in SPSS 23.0. The significance of independent and chain mediation pathways was evaluated using the bootstrap method with 5,000 resamples.

**Results:**

The results indicated that Internet use was significantly and positively correlated with cognition in older adults (*β* = 0.098, *p* < 0.001). Mediation analysis revealed a total indirect effect of 0.091 (95% CI = [0.079, 0.102]). Specifically, the indirect effect through activities of daily living was 0.029 (95% CI = [0.023, 0.036]), and the indirect effect through depression was 0.058 (95% CI = [0.049, 0.067]). Furthermore, the chain mediation effect involving both activities of daily living and depression was 0.004 (95% CI [0.003, 0.005]).

**Conclusion:**

The results indicated a significant positive correlation between Internet use and cognition in older adults. Furthermore, mediation analysis revealed that activities of daily living and depression played a partial mediating role between Internet use and cognition, involving both independent and chain mediating pathways. This suggests that Internet use may be linked to better cognitive performance through its association with higher levels of activities of daily living and lower levels of depression. Therefore, it is recommended to actively promote the digital inclusion of older adults. By providing digital skills training, enhancing Internet accessibility, and constructing age-friendly digital environments, society can help older adults better engage in the digital world, ultimately fostering their physical and mental well-being as well as active aging.

## 1 Introduction

With advancing age, the natural decline of cognitive function has evolved into a significant global public health challenge. Data from the World Health Organization (2021) indicate that the number of individuals living with cognitive impairment worldwide has exceeded 50 million (World Health Organization, 2021). The situation in China is particularly severe; as of 2020, the prevalence of dementia among older adults reached 6%, affecting approximately 15.07 million people, a figure that continues to rise (Jia et al., 2020). Cognitive impairment manifests primarily as declines in memory, visuospatial ability, and executive function (Aarsland et al., 2017). These deficits not only severely undermine functional independence and mental health but also exacerbate the burden on family caregiving and economic resources, leading to increasing pressure on healthcare systems. Therefore, maintaining cognitive health in later life is a critical measure in addressing the challenges of population aging.

Concurrently, Internet use reflects, to some extent, the digital literacy of older adults. Digital literacy, generally defined as an individual’s ability to access, understand, and utilize digital information and technology, is a vital prerequisite for participation in a digital society. Higher digital literacy enhances the capacity and willingness of older adults to engage with digital tools, thereby facilitating activities such as online information acquisition, social interaction, and the utilization of digital services. Thus, Internet use is not merely a technical behavior but also an important lifestyle factor that correlates with social participation and health status. According to the 54th Statistical Report on China’s Internet Development, the Internet penetration rate has reached 78%, indicating that digital technology has become deeply integrated into the daily lives of older adults. Previous research suggests that Internet use is positively associated with the maintenance of cognitive health (Zhou et al., 2024). Cognitive Reserve Theory posits that the brain can cope with damage through pre-existing cognitive resources. By engaging with the Internet, older adults can acquire effective health information (Wang, 2025), increase their cognitive reserve, and form more efficient cognitive networks, which may delay cognitive decline (Zhu et al., 2025). Furthermore, digital involvement represents a process of re-socialization, helping older adults learn new life skills, cultivate healthy lifestyles, and enhance their capacity for disease prevention (Quittschalle et al., 2020) while adapting to social changes (Hülür and Macdonald, 2020). Activity Theory suggests that social participation helps mitigate depression and trends of cognitive decline (Yang et al., 2025). Increasing social interaction through Internet use is considered an effective intervention for cognitive health (Jeon and Charles, 2024). However, some studies have found that the positive correlation between the Internet and cognitive function is not significant (Slegers et al., 2009), or may even be associated with negative outcomes for psychological well-being (Mu et al., 2023).

Despite numerous empirical studies exploring the relationship between Internet use and cognitive ability, several research gaps remain. First, the specific mechanisms through which the Internet relates to cognitive performance are complex, and existing literature has yet to fully elucidate these internal pathways. Second, inconsistencies in previous findings suggest a need for deeper exploration. The inconsistency in research findings may stem from overlooking complex mediating mechanisms—specifically, how Internet use is associated with the multidimensional reshaping of the aging process through both functional and psychological health. To address this gap, the present study examined the mechanisms linking Internet use and cognition among older adults from the perspective of “functional health–psychological health.” From a functional health dimension, Internet use provides “digital empowerment” (e.g., online shopping, mobile healthcare), which is linked to better activities of daily living — the foundation of cognitive health (Ren et al., 2024). From a psychological health dimension, the Internet serves a “social compensation” role, potentially alleviating loneliness and depressive symptoms, the latter being a recognized risk factor for cognitive decline (Yang et al., 2020).

Based on this rationale, the present study utilizes data from the 2023 China Longitudinal Aging Social Survey (CLASS) to construct a chain mediation model relating Internet use and cognition in older adults, exploring its mediating mechanisms through the dual dimensions of activities of daily living and depression. Compared to existing research that primarily focuses on single-factor explanatory pathways, this study systematically examines the relationship between Internet use and cognition among older adults using a nationally representative dataset. By examining the potential mechanisms linking Internet use and cognitive health from the dual perspective of activities of daily living and depression, this study provides new empirical evidence for understanding the health implications of digital technology in the context of active aging.

### 1.1 The relationship between internet use and cognition

Existing literature suggests that Internet use is associated with the stimulation of the central nervous system and neuroplastic changes, which are concurrently linked to the recovery of brain function (Sato et al., 2023). A 10-year longitudinal study found that Internet use is correlated with a significantly lower incidence of cognitive impairment among older adults in Brazil (Quialheiro et al., 2022). Substantial evidence indicates that the utilization of digital technologies, such as computers, is associated with superior memory and orientation performance in the elderly (Ramos et al., 2021). Furthermore, the positive association between Internet use and cognitive function appears more pronounced among the “young-old” group (aged 45–59), males, rural residents, and individuals with higher educational attainment (Xia et al., 2024). A vast body of research identifies Internet use as a significant protective factor for cognitive function in later life, playing a key role in its association with delayed cognitive decline and enhanced cognitive health (Jiao et al., 2025).

Accordingly, we propose the following hypothesis H1: There is a significant positive correlation between Internet use and cognition in older adults.

### 1.2 The mediating role of activities of daily living

Activities of daily living serve as a crucial indicator for assessing the physical functional status and self-care ability of older adults, directly reflecting their functional health (Zhang et al., 2021). Impairment in activities of daily living represents not only a decline in physical functioning but is also frequently associated with a heightened risk of social isolation (Kiyoshige et al., 2019). Nevertheless, the advancement of digital technologies is linked to a profound influence on the quality of daily life among older adults by facilitating social participation (Kamin et al., 2021). Research suggests that Internet use and email involvement are associated with a lower risk of impairment in instrumental activities of daily living (Sims et al., 2017). By potentially enhancing “intellectual activity” and “social role” functions, Internet use is associated with superior performance in instrumental activities of daily living and the suppression of decline in high-level functional capacity (Tajika et al., 2024). Data indicate that the incidence of impairment in activities of daily living is 13.3% lower among older adults with digital access compared to those without (Ding et al., 2023). Concurrently, activities of daily living serve as a significant factor associated with cognitive function in older adults (Ren et al., 2024). Among the Chinese elderly population, impairment in instrumental activities of daily living is significantly and negatively correlated with cognitive function. The underlying mechanism may lie in the reduction of social participation associated with declining functional capacity, which in turn correlates with a weakened cognitive state (Huang et al., 2024). Limitations in activities of daily living are closely linked to cognitive deterioration and accelerated decline (Peng et al., 2023). A decline in activities of daily living may restrict brain utilization; specifically, a lack of cognitive stimulation is associated with hippocampal atrophy, which subsequently relates to deficits in memory and emotional regulation (Ge et al., 2024). Furthermore, constraints in activities of daily living are associated with reduced daily activities and social engagement, lower levels of environmental cognitive stimulation, and an accelerated trend in cognitive decline (Li J. et al., 2025).

Based on this, we propose research hypothesis H2: Activities of daily living mediate the relationship between internet use and cognition.

### 1.3 The mediating role of depression

As a prevalent psychiatric condition, depression is associated with an increased risk of suicide and a substantial overall disease burden among older adults (Malhi and Mann, 2018). Depressive symptoms in later life are linked to a diminished quality of life and are frequently associated with cognitive decline, social isolation, and increased utilization of healthcare resources. The rapid progression of digital technologies presents new avenues for enhancing the psychological well-being of the elderly (Li and Liu, 2025). Internet use is associated with positive mental health outcomes by facilitating the maintenance of social ties, the expansion of social networks (Wang et al., 2023), the accumulation of social capital, and the promotion of social participation (Long et al., 2024). Numerous studies have confirmed that Internet use is significantly and negatively associated with depression in older adults (Ji et al., 2025), with supporting findings reported in studies from China (Wang et al., 2019) and Myanmar (Sasaki et al., 2024). Concurrently, depressive symptoms exhibit a significant negative correlation with cognitive function. Research indicates that depressive symptoms are linked to multiple cognitive dimensions, including executive function, processing speed, and memory (Brewster et al., 2017). Greater severity of depression is associated with poorer baseline memory and a more rapid rate of decline over time (Yin et al., 2024). Furthermore, severe clinical depressive symptoms serve as a risk factor associated with the accelerated deterioration of cognitive function in middle-aged and older adults (Yang et al., 2020).

Accordingly, we propose the following hypothesis H3: Depression mediates the relationship between internet use and cognition.

### 1.4 The chain mediating role of activities of daily living and depression

Activities of daily living serve as a significant factor associated with depression (Feng et al., 2021). Research indicates that a decline in activities of daily living is closely linked to depressive symptoms in older adults (He et al., 2019; Liu et al., 2023). Specifically, deficits in both basic activities of daily living and instrumental activities of daily living exhibit a positive correlation with depressive symptoms (Li et al., 2019). Individuals with lower capacity in activities of daily living, often due to the loss of independence and the requirement for long-term care, demonstrate a higher likelihood of experiencing diminished self-esteem and depressive symptoms (Sun et al., 2024). Concurrently, elevated levels of depression are associated with cognitive decline in the elderly. Older adults exhibiting depressive symptoms tend to demonstrate poorer performance in cognitive domains such as executive function, verbal memory, and information processing speed (Yin et al., 2024). Those experiencing intensified depressive symptoms in later life often exhibit the most pronounced trends of cognition decline (Formánek et al., 2020). Furthermore, the associations between Internet use and activities of daily living, depressive symptoms, and cognitive function have been supported by relevant empirical evidence (Zhu et al., 2025).

Based on this, we propose Research Hypothesis H4: Activities of daily living and depression play a chain mediating role in the relationship between internet use and cognition.

**Figure 1:**
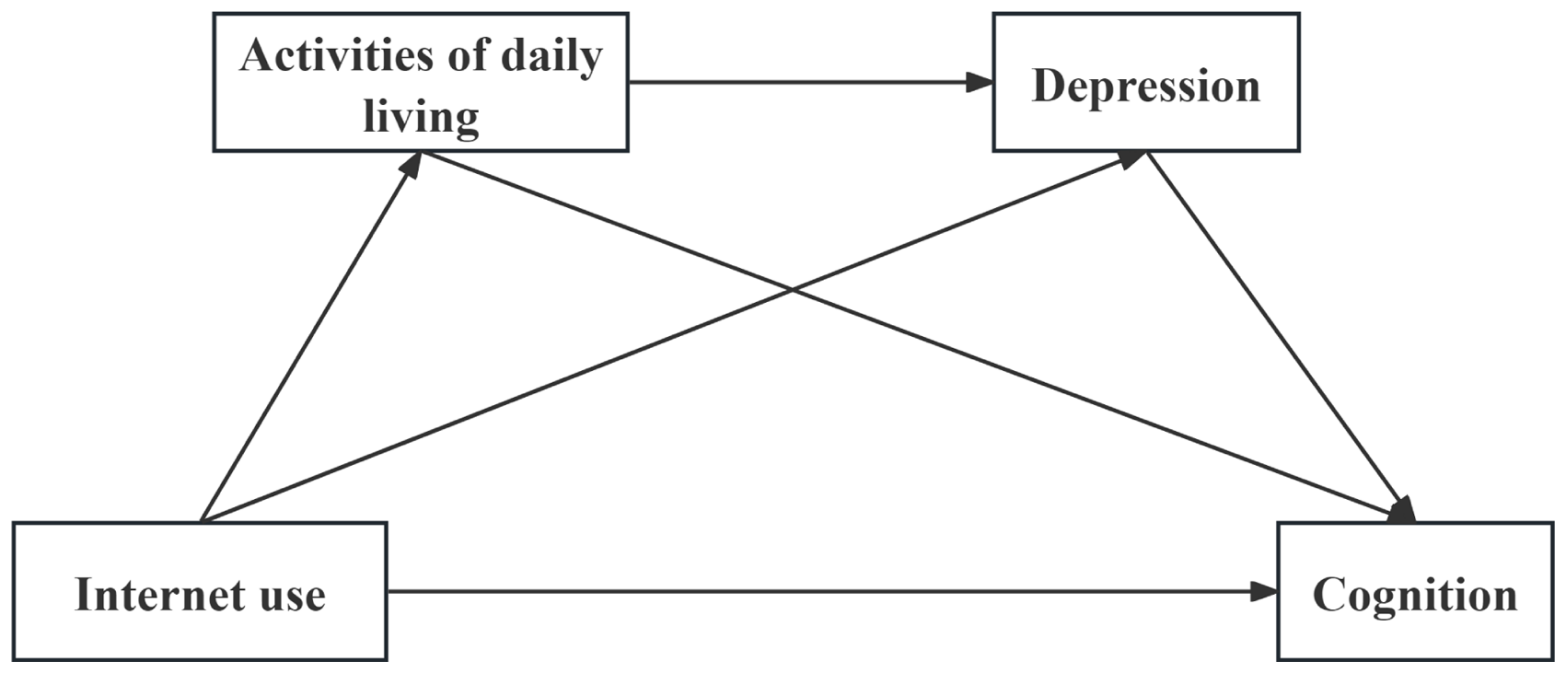
Theoretical framework of the chain mediation model.

The chain mediation model illustrating the association between Internet use and cognition in older adults is presented in Figure 1.

## 2 Materials and methods

### 2.1 Data sources

The data for this study were obtained from the 2023 wave of the China Longitudinal Aging Social Survey (CLASS), a nationally representative continuous survey project conducted by the Institute of Gerontology at Renmin University of China. Since its inception in 2014, the CLASS has completed four biennial follow-up waves in 2016, 2018, 2020, and 2023. The survey employed a stratified multi-stage probability sampling design, covering 28 provinces, autonomous regions, and municipalities across mainland China (excluding Hainan, Xinjiang, Tibet, and the Hong Kong, Macao, and Taiwan regions).

### 2.2 Variable measurement

#### 2.2.1 Internet use

The 2023 China Longitudinal Aging Social Survey (CLASS) recorded internet use of older Chinese adults. Based on previous research (Liu et al., 2024), the frequency of Internet use was measured using the item from the CLASS questionnaire: “How often have you used the Internet in the past three months?” Responses were rated on a 5-point scale (1 = never, 2 = rarely, 3 = sometimes, 4 = often, 5 = always). Higher scores indicate a higher frequency of Internet use.

#### 2.2.2 Cognition

The 2023 China Longitudinal Aging Social Survey (CLASS) was used to assess the cognitive status of older Chinese adults. In the CLASS database, cognitive function is measured using the Mini-Mental State Examination (MMSE). The questionnaire assesses five specific domains from the scale: orientation to time, orientation to place, immediate memory, delayed memory, and attention and calculation. The total score ranges from 0 to 16, with higher scores indicating better cognitive function. The validity of this scale within the older Chinese population has been confirmed by multiple studies (Zhu et al., 2025). In the present study, the Cronbach’s *α* coefficient for the scale was 0.814.

#### 2.2.3 Activities of daily living

Activities of daily living comprise basic activities of daily living and instrumental activities of daily living. In the 2023 China Longitudinal Aging Social Survey (CLASS) questionnaire, basic activities of daily living were assessed using eight indicators: dressing, bathing, eating, bowel control, bladder control, toileting, transferring between bed and chair, and indoor mobility. Instrumental activities of daily living were assessed using eight indicators: making phone calls, walking up and down stairs, grooming, walking outdoors, using public transportation, shopping, doing housework, and cooking. Each item is rated on a three-point scale (1 = completely dependent, 2 = partially dependent, 3 = completely independent). The total score is the sum of scores from both basic activities of daily living and instrumental activities of daily living, ranging from 16 to 48. Higher scores indicate greater capability in activities of daily living. The validity of this scale within the older Chinese population has been confirmed by multiple studies (Yan et al., 2025). In the present study, the Cronbach’s *α* coefficient for the scale was 0.937.

#### 2.2.4 Depression

The 2023 China Longitudinal Aging Social Survey (CLASS) recorded individual depression status, using the Center for Epidemiologic Studies Depression Scale (CES-D) to assess depressive symptoms in older adults. The scale consists of nine items covering a range of emotional states and physiological symptoms associated with depression. Specifically, the scale measures six negative emotions and physiological manifestations: feelings of loneliness, sadness, loss of appetite, sleep disturbance, feelings of worthlessness, and a sense of boredom; it also assesses three positive emotions: happiness, life satisfaction, and enjoyment. Items on the CES-D are rated on a 3-point scale (1 = never, 2 = sometimes, 3 = often). To ensure the scale focuses on measuring depressive symptoms, items representing positive emotions are reverse-scored. The total score is calculated by summing the scores of the nine items, resulting in a composite index ranging from 9 to 27. Higher scores indicate more severe depressive symptoms. The validity of this scale within the older Chinese population has been confirmed by multiple studies (Zhu et al., 2025). In the present study, the Cronbach’s α coefficient for the scale was 0.726.

#### 2.2.5 Control variables

Based on existing literature (Huang et al., 2024), gender, age, education level, and income level were selected as control variables. Variable definitions and descriptive statistics are presented in Table 1.

**Table 1:** Variable definitions and descriptive statistics.

Variable type	Variables	Variable declaration	Mean	SD	Min	Max
Dependent variable	Cognition	—	13.642	2.712	0	16
Independent variable	Internet use	1 = Never use; 2 = Rarely use; 3 = Sometimes use; 4 = Often use; 5 = Always use	2.539	1.913	1	5
Mediating variable	Activities of daily living	1 = Completely dependent; 2 = Partially dependent; 3 = Fully independent	47.416	2.169	16	48
	Depression	1 = Never; 2 = Sometimes; 3 = Often	15.584	3.332	9	27
Control variables	Gender	0 = Female; 1 = Male	0.518	0.499	0	1
	Age	Actual age of respondents at the time of the survey	71.299	5.815	60	90
	Education	0 = No education; 1 = Elementary school; 2 = Junior high school; 3 = High school; 4 = College or above	1.364	0.962	0	4
	Income	Logarithm of Total Annual Revenue for 2022	9.507	1.173	4.606	12.899

### 2.3 Statistical analysis

Data were cleaned using Stata 18.0, and regression analysis was conducted to examine the relationship between Internet use and cognition (H1). To test the mediating mechanisms between Internet use and cognitive function in older adults, the PROCESS macro for SPSS (Version 23.0, Model 6) was utilized. A bootstrapping procedure with 5,000 resamples was conducted to assess the mediating roles of activities of daily living (H2) and depression (H3), as well as the chain mediating pathway involving both activities of daily living and depression (H4). Prior to the regression analysis, multicollinearity tests were conducted. The results indicated that the Variance Inflation Factor (VIF) values for all variables ranged from 1.02 to 1.47 (all below the threshold of 5), suggesting that no severe multicollinearity issues were present in the models. Furthermore, the basic assumptions of regression analysis—including linearity, homoscedasticity, and normality of residuals—were evaluated, and no significant violations were observed.

## 3 Results

### 3.1 Correlation analysis of key variables

The results of the correlation analysis for key variables are presented in Table 2. Specifically: (1) Internet use was significantly and positively correlated with both activities of daily living (*r* = 0.138, *p* < 0.001) and cognition (*r* = 0.227, *p* < 0.001); (2) Internet use was significantly and negatively correlated with depression (*r* = −0.316, *p* < 0.001); (3) activities of daily living were significantly and negatively correlated with depression (*r* = −0.165, *p* < 0.001) and positively correlated with cognition (*r* = 0.232, *p* < 0.001); and (4) depression was significantly and negatively correlated with cognition (*r* = −0.262, *p* < 0.001).

**Table 2:** Correlation analysis of key variables.

Variables	Internet use	Activities of daily living	Depression	Cognition
Internet use	1			
Activities of daily living	0.138^***^	1		
Depression	−0.316^***^	−0.165^***^	1	
Cognition	0.227^***^	0.232^***^	−0.262^***^	1

*p < 0.05, **p < 0.01, ***p < 0.001.

### 3.2 The association between internet use and cognition

A multiple linear regression model was constructed with cognition as the dependent variable, Internet use as the independent variable, and selected covariates as control variables (Table 3). In Model 1, which included control variables associated with cognition in older adults, the results indicated a significant negative correlation between age and cognition, suggesting that cognitive performance tends to be lower at advanced ages. Furthermore, gender, educational attainment, and income level were significantly associated with cognition. Model 2 incorporated Internet use based on Model 1. The results demonstrated a significant positive correlation between Internet use and cognition among older adults, with a regression coefficient for Internet use of 0.189 (p < 0.001). This indicates that higher frequencies of Internet use are concurrently linked to superior levels of cognitive performance. Consequently, these findings support a significant positive correlation between Internet use and cognitive function in older adults, thereby providing empirical support for Hypothesis 1.

**Table 3:** Results of multiple linear regression analysis.

Variables	Model 1 (Cognition)	Model 2 (Cognition)
Gender	−0.183^***^(0.053)	−0.190^***^(0.053)
Age	−0.077^***^(0.005)	−0.060^***^(0.005)
Education	0.335^***^(0.029)	0.261^***^(0.030)
Income	0.144^***^(0.023)	0.084^***^(0.024)
Internet use		0.189^***^(0.019)
Constant	17.703^***^(0.449)	16.719^***^(0.457)
$R^{2}$	0.072	0.083
Adj. $R^{2}$	0.071	0.082
Observations	9,012	9,012

*p < 0.05, **p < 0.01, ***p < 0.001.

### 3.3 Robustness checks

To verify the robustness of the main findings, a series of additional tests were conducted. First, the core explanatory variable was replaced to re-examine the relationship between Internet use and cognition among older adults. Specifically, the original measure of “frequency of Internet use” was substituted with a binary indicator derived from the CLASS questionnaire item, “Do you use the Internet?”, where non-use was coded as 0 and use as 1. As shown in Model 3 of Table 4, Internet use remains significantly and positively associated with cognition among older adults, with statistical significance at the 0.001 level. Second, an alternative estimation method was employed as a further robustness check. An ordered Logit model was used to re-estimate the relationship between Internet use and cognition. The results, reported in Model 4 of Table 4, continue to indicate a significantly positive association between Internet use and cognition among older adults, again at the 0.001 significance level. These results further confirm the robustness of the main conclusions of this study.

**Table 4:** Robustness check results.

Variables	Model 3 (Cognition)	Model 4 (Cognition)
Gender	−0.187^***^(0.053)	−0.108^**^(0.038)
Age	−0.067^***^(0.005)	−0.044^***^(0.004)
Education	0.289^***^(0.030)	0.186^***^(0.022)
Income	0.108^***^(0.024)	0.061^***^(0.017)
Internet use	0.379^***^(0.062)	0.125^***^(0.014)
Constant	17.236^***^(0.455)	—
$R^{2}$	0.076	—
Adj. $R^{2}$	0.075	—
Observations	9,012	9,012

*p < 0.05, **p < 0.01, ***p < 0.001.

### 3.4 Testing the chain mediation effect

The mediation effect was tested using the PROCESS macro (Model 6) for SPSS 23.0 developed by Hayes. A bootstrap method with 5,000 resamples was employed to estimate the 95% confidence intervals (CI) for the effects. Gender, age, education level, and income were controlled for in all analyses.

**Figure 2:**
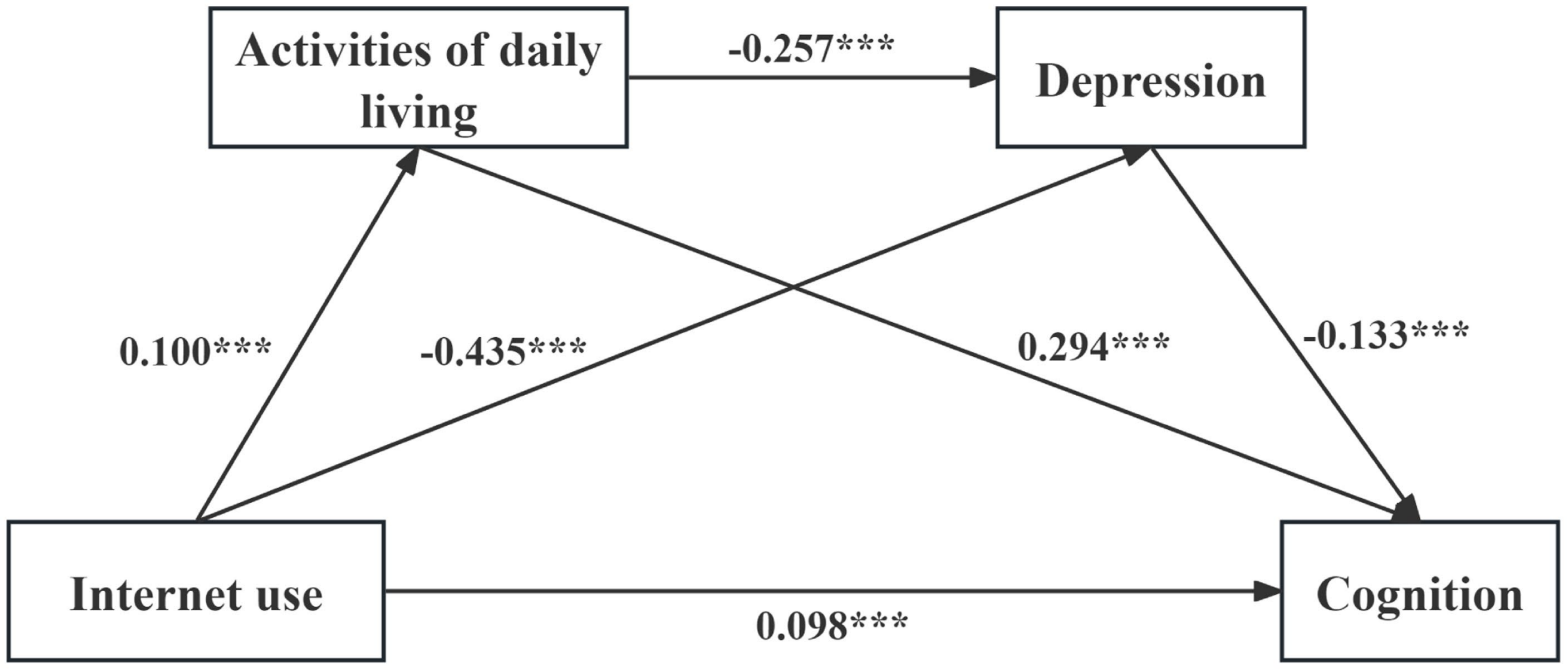
Chain mediation model of internet use and cognition. *p < 0.05, **p < 0.01, ***p < 0.001.

The results of the chain mediation regression analysis are presented in Table 5. Internet use was significantly and positively associated with activities of daily living (*β* = 0.100, *p* < 0.001). Furthermore, both Internet use and activities of daily living were significantly and negatively associated with depression (*β* = −0.435, *p* < 0.001; *β* = −0.257, *p* < 0.001, respectively). After including activities of daily living and depression as mediating variables, Internet use remained significantly and positively associated with cognition (*β* = 0.098, *p* < 0.001). Meanwhile, activities of daily living showed a significant positive association with cognition (*β* = 0.294, *p* < 0.001), while depression exhibited a significant negative association with cognition (*β* = −0.133, *p* < 0.001). These findings indicate that activities of daily living and depression play a partial mediating role in the relationship between Internet use and cognition in older adults. In other words, Internet use was not only directly associated with cognition in older adults but was also indirectly associated with cognition through activities of daily living and depression (see Figure 2 for the chain mediation model).

**Table 5:** Regression analysis results of variable relationships in the mediation model.

Variables	Model 5 (activities of daily living)	Model 6 (depression)	Model 7 (cognition)
Gender	−0.074^*^(0.032)	0.178^**^(0.065)	−0.142^**^(0.051)
Age	−0.033^***^(0.003)	0.033^***^(0.006)	−0.045^***^(0.005)
Education	0.017(0.018)	−0.238^***^(0.037)	0.224^***^(0.029)
Income	−0.105^***^(0.015)	−0.226^***^(0.029)	0.088^***^(0.023)
Internet use	0.100^***^(0.011)	−0.435^***^(0.023)	0.098^***^(0.018)
Activities of daily living		−0.257^***^(0.021)	0.294^***^(0.017)
Depression			−0.133^***^(0.008)
Constant	50.805^***^(0.277)	28.797^***^(1.218)	3.873^***^(0.990)
$R^{2}$	0.038	0.132	0.143
Adj. $R^{2}$	0.037	0.132	0.142
Observations	9,012	9,012	9,012

*p < 0.05, **p < 0.01, ***p < 0.001.

Furthermore, Model 7 accounted for 14.3% of the variance (R^2^ = 0.143) in cognition. This moderate explanatory power is likely due to the fact that, within large-sample social survey data, older adults cognitive function is shaped by a complex interplay of physiological, psychological, and socio-environmental factors. Despite being moderate, this proportion of explained variance remains reasonable, holds explanatory value, and is highly consistent with related research (Jiao and Sun, 2025).

The results of the chain mediation effect test are shown in Table 6. The total effect of Internet use on cognition in older adults was 0.189, with a 95% CI of [0.152, 0.225], which did not contain 0. After including the mediating variables of activities of daily living and depression, the direct effect of Internet use on cognition was 0.098, with a 95% CI of [0.062, 0.134], which did not contain 0. The total indirect effect of the mediators (activities of daily living and depression) was 0.091, with a 95% CI of [0.079, 0.102], which did not contain 0, accounting for 48.15% of the total effect. Specifically, the indirect effect via activities of daily living was 0.029 (95% CI = [0.023, 0.036], excluding 0), accounting for 15.34% of the total effect; thus, Hypothesis 2 was supported. The indirect effect via depression was 0.058 (95% CI = [0.049, 0.067], excluding 0), accounting for 30.69% of the total effect; thus, Hypothesis 3 was supported. The chain mediating effect via activities of daily living and depression was 0.004 (95% CI = [0.003, 0.005], excluding 0), accounting for 2.12% of the total effect; thus, Hypothesis 4 was supported.

**Table 6:** Bootstrap test results for the chain mediation model.

Path	Effect size	Boot SE	Boot 95% CI LLCI	Boot 95% CI ULCI	Percentag e(%)
Total effect	0.189	0.018	0.153	0.225	100%
Direct effect	0.098	0.018	0.062	0.134	51.85%
Total indirect effect	0.091	0.005	0.079	0.102	48.15%
Internet use → activities of daily living → cognition	0.029	0.003	0.023	0.036	15.34%
Internet use → depression → cognition	0.058	0.004	0.049	0.067	30.69%
Internet use → activities of daily living → depression → cognition	0.004	0.0004	0.003	0.005	2.12%

## 4 Discussion

### 4.1 The association between internet use and cognition among older adults

The findings indicated that Internet use was significantly and positively associated with cognitive function in older adults, which is consistent with previous research (Kamin and Lang, 2020). For instance, lower frequencies of computer use in cognitively healthy older adults have been linked to reduced hippocampal volume, a critical region for cognitive maintenance (Silbert et al., 2016). As a complex networked system, the Internet involves various executive functions, such as attentional allocation, information filtering, working memory updating, and decision-making (Yuan et al., 2022). Such sustained digital engagement may be related to increased synaptic density and the activation of key brain regions, which has been associated with better cognitive performance (Small et al., 2009). Furthermore, Internet use may broaden information channels for older adults and may be associated with continued social interaction, which in turn may relate to a lower risk of cognitive decline linked to the weakening of social roles (Zhu et al., 2025). From the perspective of social participation theory, Internet use exhibits a compensatory association with the “structural disengagement” related to physical decline. Furthermore, participation in online social interactions and interest groups is not only accompanied by the reconstruction of older adults social networks but is also significantly and positively associated with the preservation of brain function (Liu et al., 2025). Given the brain’s plasticity in response to environmental stimuli, this rich and sustained digital participation may represent an important factor associated with the maintenance of cognitive reserve. Overall, as a highly accessible form of activity, Internet use was significantly and positively associated with cognitive performance in older adults.

### 4.2 The mediating role of activities of daily living

The results indicated that activities of daily living exerted a significant independent mediating effect on the relationship between Internet use and cognition in older adults. This indicates that Internet use is not only directly associated with cognitive function but also forms an indirect pathway through activities of daily living. As a digital resource, Internet use may support older adults in managing daily tasks—such as online shopping, medical appointments, and mobile payments—and may be associated with better environmental adaptability and task-management capacity (Li et al., 2022). These findings lend support to the perspectives proposed in previous research (Peng et al., 2023). Furthermore, seeking health information via the internet is associated with improved health literacy, which in turn is linked to a healthier lifestyle and higher levels of physical functioning (Li et al., 2024). Furthermore, the level of activities of daily living is significantly and positively associated with proficiency in basic life skills. It is also interconnected with higher levels of physical activity and social participation, all of which are closely linked to better cognitive performance (Jeon et al., 2022). Recent empirical evidence has also confirmed that interventions targeting instrumental activities of daily living yield significant improvements in overall cognition (Li W. et al., 2025). Accordingly, activities of daily living may represent an important pathway through which Internet use is associated with cognitive function.

### 4.3 The mediating role of depression

The findings revealed that depression serves as a significant independent mediator between Internet use and cognition, suggesting that Internet use is indirectly linked to cognitive performance through mental health factors. Prior research has extensively established a significant negative correlation between depressive symptoms and cognitive function (Muhammad and Meher, 2021). A systematic review and meta-analysis indicated that both clinical and subclinical depression are associated with deficits in cognitive control (Dotson et al., 2020). Chronic depression demonstrates an association with hypothalamic–pituitary–adrenal(HPA) axis dysregulation and elevated glucocorticoid levels, a physiological state that is accompanied by signs of neuronal damage in the hippocampus (Sawyer et al., 2012). Furthermore, this state exhibits a significant negative correlation with memory and executive functioning (D'Alessio et al., 2020). Meanwhile, Internet use was significantly and negatively associated with depressive symptoms among older adults. The Internet may provide an important channel for social engagement among older adults. Through instant messaging, video calls, and social media interaction, older adults may maintain contact with family members and broader social networks, which has been associated with lower levels of loneliness and social isolation (Hu et al., 2025). Social support has been shown to be significantly and positively associated with mental health among older adults, while online social interaction may partially compensate for the emotional loss associated with reduced opportunities for offline interpersonal contact (Cheng et al., 2024). Furthermore, the sense of engagement and self-efficacy derived from online leisure and learning activities is associated with lower levels of negative affect (Craig et al., 2022). Accordingly, depression may represent a key psychological pathway through which Internet use is associated with cognitive function.

### 4.4 The chain mediating effect of activities of daily living and depression

The study further identified a significant chain mediation effect among internet use, activities of daily living, depression, and cognitive function. Specifically, higher levels of Internet use were associated with higher levels of activities of daily living, which in turn were associated with lower levels of depressive symptoms and, subsequently, with better cognitive performance. A higher level of activities of daily living generally indicates greater independence and a sense of control in real life for older adults, a state that is linked to a lower risk of depression (Redzovic et al., 2023). Meanwhile, the level of functional independence in older adults is significantly and positively associated with the frequency of social participation and outdoor activities (Beltz et al., 2022). This elevated activity level is significantly and positively correlated with the breadth of social networks, while being significantly and negatively correlated with negative emotional levels (Liu et al., 2023). Furthermore, a positive emotional state is significantly and positively associated with more optimal attention allocation and higher information processing efficiency, and significantly and negatively associated with the extent to which negative emotions occupy limited cognitive resources (Fredrickson and Branigan, 2005). Additionally, multiple studies indicate that intense or persistent levels of negative emotions are positively associated with the consumption of limited attentional resources, and significantly and negatively correlated with cognitive performance areas such as working memory, prospective memory, and arithmetic processing (Lallement et al., 2025). Overall, this study elucidates the interconnected pathways between Internet use and cognition among older adults, offering a multidimensional perspective for understanding the complex relationship between Internet use and cognition.

### 4.5 Theoretical contributions

Building upon existing literature, this study extends the research on the relationship between Internet use and cognitive health in older adults. First, this research shifts the focus from isolated factors to an integrated “functional health–mental health” perspective. Unlike many previous studies that examined the association between Internet use and cognitive function through a single lens—such as social interaction or mental health—this study incorporates both activities of daily living and depression into a unified analytical framework. By examining multi-level associative pathways linking Internet use and cognitive function, this study provides a more integrated basis for understanding how digital engagement relates to aging across functional and psychological health.

Second, while previous mediation studies have predominantly focused on single-factor explanatory pathways, this study provides an incremental contribution by testing a chain mediation model of “Internet use–activities of daily living–depression–cognition.” The findings indicate that Internet use is associated with cognitive function in older adults both directly and indirectly through a chained pathway. Specifically, Internet use is associated with higher levels of activities of daily living, which in turn are linked to lower levels of depression, and subsequently correlate with better cognitive performance. This chain mechanism offers a more comprehensive theoretical explanation of the interrelationships among digital participation, functional health, and psychological well-being in later life.

### 4.6 Practical implications

The present findings offer significant policy implications for promoting cognitive health and supporting healthy aging among older adults. First, enhancing internet accessibility and digital literacy among the elderly is crucial to bridging the digital divide between older and younger populations. Governments and community organizations can facilitate this process by implementing targeted digital literacy programs and establishing community-based digital learning platforms, thereby enabling older adults to acquire essential internet skills and strengthen both information access and social engagement. Second, the development of age-friendly digital products and services should be prioritized. Internet platforms and digital service providers need to account for the specific needs of older users by simplifying interface design, optimizing font size and visual layout, and incorporating voice-assisted functionalities. Such adaptations may improve the usability and accessibility of digital tools and may help older adults navigate online services more efficiently and independently. Third, integrating internet-based applications into healthy aging interventions represents a promising strategy. Community and eldercare organizations can leverage online platforms to deliver health education, provide telemedicine consultations, and facilitate virtual social activities. These initiatives may support social participation and psychological well-being and may also be relevant to strategies aimed at maintaining cognitive health in older adults.

### 4.7 Limitations and future perspectives

Several limitations should be acknowledged. First, the 2023 China Longitudinal Aging Social Survey (CLASS) dataset used in this research is cross-sectional in nature. Although it identified associations and potential mediating pathways between Internet use and cognition in older adults, causal inferences cannot be drawn. While the chain mediation model possesses a clear theoretical rationale, the cognitive level of older adults may also reversely affect their Internet use skills and frequency, suggesting a potential bidirectional relationship. Furthermore, although this study controlled for a range of demographic variables, potential endogeneity issues driven by unobserved confounding factors (e.g., individual cognitive reserve and early education quality) may still exist. Therefore, future studies should use longitudinal data or quasi-experimental designs to further clarify the directionality and causal nature of this relationship. Second, internet use was measured with a single frequency item, without distinguishing types or purposes of online activity, such as information seeking, social interaction, or entertainment. Different activities may have distinct cognitive effects. Future studies should adopt multidimensional measures to capture these variations. Third, cognitive function was assessed using a brief MMSE (0–16), which may not detect high-level cognitive differences. More comprehensive tools, such as multidimensional cognitive batteries or longitudinal indicators, are recommended. Fourth, the scores for activities of daily living were generally high in this sample, potentially causing ceiling effects that limit variability and affect the stability of the estimates. Differentiating functional groups or employing finer-grained measures may improve the accuracy of future empirical findings.

Despite these limitations, our study possesses several notable strengths. First, it uses the latest nationally representative 2023 CLASS data, providing updated evidence on older adults internet use and cognitive function in China’s rapidly digitizing context. Second, this study incorporates activities of daily living and depression into a unified analytical framework, examining the multi-level associative mechanisms between Internet use and cognitive performance. This approach provides a more comprehensive view of the complex interrelationships among factors related to cognitive health in older adults. Third, findings offer empirical support for promoting digital inclusion and healthy aging, with practical implications for policies on digital access and aging health.

## 5 Conclusion

Using data from the 2023 China Longitudinal Aging Social Survey (CLASS), this study examined the association between Internet use and cognitive function among older adults, as well as the underlying mediating mechanisms. The main findings are summarized as follows:

(1) Internet use is positively associated with cognitive function in older adults. (2) Both activities of daily living and depression partially mediate the relationship between internet use and cognitive function. (3) Activities of daily living and depression play a chain mediating role in the relationship between Internet use and cognitive function in older adults.

## Data Availability

Publicly available datasets were analyzed in this study. This data can be found at: http://class.ruc.edu.cn.
